# Key influencing factors analysis on life satisfaction among Chinese older adults with hypertension: a National Cross-Sectional Survey

**DOI:** 10.3389/fpubh.2025.1569935

**Published:** 2025-04-28

**Authors:** Yazhu Wang, Ziyi Chen, Heqian Fan, Shiwei Cao, Xiaoyu Wang, Tengfei Niu

**Affiliations:** ^1^Department of Cardiology, The Shapingba Hospital, Chongqing University (People’s Hospital of Shapingba District), Chongqing, China; ^2^College of Traditional Chinese Medicine, Chongqing Medical University, Chongqing, China; ^3^College of Pediatric, Chongqing Medical University, Chongqing, China; ^4^The Second Clinical College, Chongqing Medical University, Chongqing, China; ^5^Department of Basic Courses, Chongqing Medical and Pharmaceutical College, Chongqing, China

**Keywords:** hypertension, life satisfaction, older adults, CLHLS, Cross-Sectional Survey

## Abstract

**Objectives:**

This study aimed to assess the current situation and influencing factors of life satisfaction among Chinese older adults with hypertension and to identify its key factors.

**Methods:**

In this study, 4,197 hypertensive patients were selected from the Chinese Longitudinal Healthy Longevity Survey (CLHLS) database for inclusion in the analysis. A multivariate logistic regression model was used to analyze the influencing factors of life satisfaction in hypertensive patients, and the random forest was further used to rank the importance of the significant influencing factors.

**Results:**

Overall, 29.52% of hypertensive patients reported dissatisfaction with their lives. The life satisfaction of these patients was influenced by a combination of factors. According to the results of the random forest, the variables that significantly influenced life satisfaction, in descending order of importance, are self-rated health, economic status, depressive symptoms, sleep duration, fruits, living arrangements, hearing impairment, heart disease, and gender.

**Conclusion:**

Our research indicates that currently, people with hypertension experience a high level of dissatisfaction with their lives, making it necessary to take preventive and intervention measures from multiple aspects.

## 1 Introduction

Population aging has inevitably become a major issue commonly faced across the globe (1). Studies have shown that the aging level in China has ranked among the upper-middle tier globally, with unprecedented speed and scale (2).

The aging population presents a heavy burden of diseases. Among these, hypertension, being the most common cardiovascular disease, occupies an important position (3). Currently, there are more than 1 billion adults with hypertension worldwide (4), and it is estimated that the global prevalence will reach 1.5 billion by 2025 (5). According to the results released in the Annual Report on National Center for Cardiovascular Diseases (6), the prevalence of hypertension among adults in China had reached as high as 31.6%. Although hypertension is one of the most preventable cardiovascular diseases and a key manageable risk factor for mortality (7), the World Health Organization (WHO) estimates that in 2019, more than half of all cardiovascular-related deaths could still be attributed to uncontrolled hypertension. Among people aged 30–79 with hypertension, only 21% have their blood pressure under control (8). Hypertension can lead to functional or organic damage to organs such as the heart, brain, and kidneys, leading to various chronic diseases like stroke, coronary heart disease, heart failure, cognitive impairment, and chronic kidney disease (9–12). All of these health issues are related to lower life satisfaction (LS) for patients (13, 14). This suggests that further understanding of the epidemiological trends and clinical features of low LS in hypertensive patients will help to improve the quality of their lives by early identification of those at high risk for low LS.

LS is defined as an individual’s subjective perception and feelings about their current state of life or overall quality of life (15, 16). It can influence an individual’s emotional experience and lifestyle, which, in turn, affects the overall atmosphere and the development of society. Numerous previous studies have reported various factors that influence the LS of older adults. For example, research conducted by Li et al. (17) found that financial pressure, depressive symptoms, filial piety, and access to medical services were significantly related to the LS of older adults. Other studies have also emphasized the significant impact of social activities (e.g., social isolation and basic community services) (18, 19) and lifestyles (e.g., exercise, diet, and sleep) (20, 21) on their LS. It is undeniable that these meaningful studies have all made important contributions to improving the LS of older adults. However, the applicability of these research conclusions to the population of older adults with hypertension in China remains to be further discussed. In addition, previous studies mainly relied on traditional logistic regression model for their research. As a machine learning algorithm, random forest (RF) is an excellent clinical research tool due to its strong classification capability and straightforward learning mechanism. In recent years, the RF algorithm has been widely used in the medical field for disease diagnosis and classification (22), clinical outcome prediction (23), and estimating the importance of exposure to risk factors (24).

In summary, this study aimed to use the cross-sectional data published by the Chinese Longitudinal Healthy Longevity Survey (CLHLS) in 2018 to analyze the influencing factors of LS in older adults with hypertension in China from the three dimensions of sociodemographic characteristics, health status, and lifestyles, and further rank the importance of these factors using the RF algorithm. The results of the study are of great reference value for intervening and improving the LS of older adults with hypertension and promoting their overall mental health.

## 2 Materials and methods

### 2.1 Data source

The data in this study came from CLHLS, and all data and questionnaire contents can be downloaded by registering on their official website.^1^ CLHLS is a national longitudinal survey project organized by the Center for Healthy Aging and Development/National School of Development of Peking University. It targeted older adults aged 65 and above and their children aged 35–64 and adopted the multi-stage whole cluster random sampling method to randomly select 631 cities and counties in 23 provinces in China, with samples covering about 85% of China’s population (25). It collected data on various aspects of the participants’ socioeconomic background, family structure, health status, and lifestyles with a high degree of reliability and representativeness. The baseline survey of the project started in 1998, and follow-up visits were conducted every 3–4 years after this. The project has provided a large amount of scientific evidence for geriatric health research, policy formulation, and social services. The CLHLS study received ethical approval from the Peking University Institutional Review Board (IRB00001052-13074), and each subject or their legal representative signed a written informed consent form.

**Figure 1:**
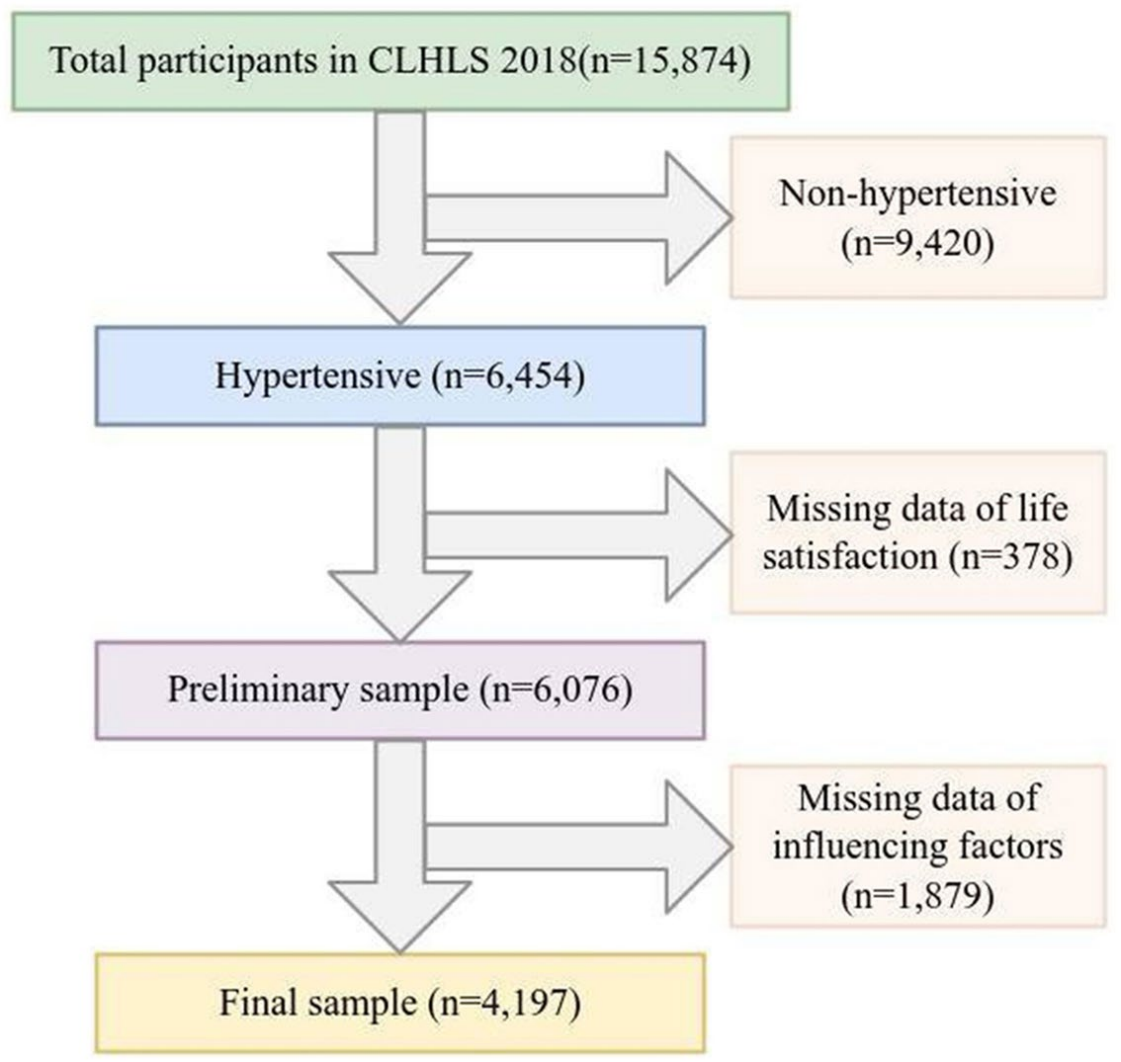
Flowchart of sample cleaning.

This study used survey data released by CLHLS in 2018 for analysis. The selection of the study sample was based on the question in the questionnaire: “Have you been diagnosed with hypertension by a doctor?” (26). Participants who answered “yes” were defined as hypertensive patients and further included in our follow-up study. In addition, after excluding samples with missing influencing factors and LS, 4,197 participants were included in the statistical analysis as valid samples. The detailed sample screening process is shown in Figure 1.

The minimum sample size required for the study was determined according to the formula for calculating sample size in cross-sectional studies:


$$N=\left(Z_{\alpha /2}^{2}pq\right)/\delta^{2}$$

(1) N denotes the sample size required for the study. (2) p denotes the prevalence of life dissatisfaction among Chinese older adults. (3) q = (1-p). (4) Z_α/2_ was set to 1. 96, and α was set to 0.05 for the two-sided test. (5) δ denotes the permissible error, calculated as 0.1p. According to the results of a previous study, the prevalence of life dissatisfaction among stroke patients in China was 16.9% (27). It was calculated that at least 1,889 participants were needed in this study to achieve the required sample size. Both stroke and hypertension are cardiovascular diseases that pose a high risk to Chinese older adults’ health. Therefore, we believe that there is a certain degree of reliability in our use of the reported rate of life dissatisfaction among stroke patients to estimate the prevalence of life dissatisfaction among hypertensive patients in this study.

### 2.2 Independent variable

Based on previous research results and the availability of the CLHLS database (28), we considered 30 influencing factors from three dimensions: sociodemographic characteristics, health status, and lifestyles. Specifically, basic demographic characteristics include age, gender, residence, marital status, education level, and economic status. Health status includes body mass index (BMI), abdominal obesity, self-rated health, visual impairment, hearing impairment, cognitive impairment, diabetes, heart disease, chronic nephritis, hepatitis, cancer, activities of daily living (ADL) disability, anxiety, and depressive symptoms. Lifestyles include fruits, vegetables, taste, sleep duration, smoking, drinking, exercise, physical activity, social participation, and living arrangements. Detailed information on the assignment of all variables is provided in Table 1. For some influencing factors with complex measurement methods, the details are as follows:

**Table 1:** Assignment of independent variables.

Dimensions	Variables	Assignment
Sociodemographic characteristics	Age	Continuous variable
	Gender	0 = female;1 = male
	Residence	0 = rural;1 = urban
	Marital status	0 = unmarried;1 = married
	Education level	0 = 0;1 = 0–6;2= > 6
	Economic status	0 = good;1 = common;2 = poor
Health status	BMI	0 = 18.5–24;1 = <18.5;2 = 24–28;3 = ≥28
	Abdominal obesity	0 = no;1 = yes
	Self-rated health	0 = good;1 = common;2 = poor
	Visual impairment	0 = no;1 = yes
	Hearing impairment	0 = no;1 = yes
	Cognitive impairment	0 = no;1 = yes
	Diabetes	0 = no;1 = yes
	Heart disease	0 = no;1 = yes
	Chronic nephritis	0 = no;1 = yes
	Hepatitis	0 = no;1 = yes
	Cancer	0 = no;1 = yes
	ADL disability	0 = no;1 = yes
	Anxiety symptoms	0 = no;1 = yes
	Depressive symptoms	0 = no;1 = yes
Lifestyles	Fruits	0 = not eating;1 = eating;2 = almost every day
	Vegetables	0 = not eating;1 = eating;2 = almost every day
	Taste	0 = not light;1 = light
	Sleep duration	0 = 7–9;1 = <7;2= > 9
	Smoking	0 = no;1 = yes
	Drinking	0 = no;1 = yes
	Exercise	0 = no;1 = yes
	Physical activity	0 = no;1 = yes
	Social participation	0 = no;1 = yes
	Living arrangements	0 = living with family members;1 = living alone;2 = living in a nursing home

BMI, body mass index; ADL, activities of daily living.

Through the formula BMI = weight (kg)/[height (m)]^2^ to calculate BMI to define body obesity, BMI < 18.5 was defined as underweight, 18.5 ≤ BMI < 24 was normal, 24 ≤ BMI < 28 was overweight, and BMI ≥ 28 was obesity. According to previous studies, men with a waist circumference of >85 cm and women with a waist circumference of >80 cm were defined as having abdominal obesity (29). The investigator shone a flashlight at the circle on the eye chart, and the participants were asked to answer whether they could see the circles and distinguish the direction of the notches. There were four options: (1) can see and distinguish. (2) can see but cannot distinguish. (3) cannot see clearly. (4) Blind. If respondents chose (2)–(4), they were assessed as visual impairment (24). Cognitive impairment was assessed by the Mini-Mental State Examination (MMSE) (30). Specifically, the scale consists of 24 questions with a total score ranging from 0 to 30, with higher scores indicating better cognitive functioning. The MMSE cut-off values are set at 16/17 for illiterate individuals, 19/20 for individuals with 1–6 years of education, and 23/24 for individuals with 7 or more years of education. After adjusting for education level, a MMSE score below the cut-off value was defined as cognitive impairment (31). The Katz Activities of Daily Living scale was used to evaluate the ADL for older adults. It includes bathing, dressing, feeding, going to the toilet, moving from bed to chair, and controlling urination and defecation. Failure to independently complete any of those was considered an ADL disability (32). Anxiety symptoms were assessed using the 7-item Generalized Anxiety Disorder (GAD-7) scale, which consists of 7 items and has a total score range of 0 to 21. A higher score indicates more severe anxiety symptoms. Individuals with a GAD-7 score >5 were classified as having anxiety symptoms (33). The 10-item Center for Epidemiological Studies Depression (CESD-10) scale was used to assess depressive symptoms in older adults. The scale consists of 10 items, with a total score ranging from 0 to 30. Individuals with a depression score of ≥10 were defined as having depressive symptoms (34). Respondents were asked whether they were involved in social activities, and their responses were classified as “yes” or “no.” Social participation was defined as the presence or absence of such participation (35).

### 2.3 Life satisfaction

LS was assessed by the question, “How satisfied are you with your life as a whole now?” (27). The answers included five options: (1) Very satisfied. (2) Satisfied. (3) Average. (4) Dissatisfied. (5) Very dissatisfied. (1), (2), and (3) were combined as satisfied as the more positive responses to LS, and the more negative responses (4) and (5) were combined as dissatisfied. LS represents an individual’s evaluation of the overall state and quality of life, and it has been widely used currently (15, 27).

### 2.4 Statistical analysis

Kolmogorov–Smirnov test was used to assess the normality of continuous variables. When *p* > 0.05, the distribution of continuous variables was considered to conform to a normal distribution. Bartlett test was used to assess the homogeneity of variance of continuous variables. When *p* > 0.05, it was considered to satisfy the homogeneity of variance. Continuous variables that conformed to normality were described by mean and standard deviation (M ± SD), and categorical variables were expressed as frequencies and percentages [n(%)]. After testing for normalcy and variance homogeneity, two independent sample t-tests were used to compare the differences in continuous variables between different levels of LS. The Chi-square test was used to compare the differences in categorical variables between different levels of LS. A multivariate logistic regression model was used to analyze the relationship between each variable and LS. We report the odds ratio (OR) value and 95% confidence interval (CI) for all variables. To further determine the importance of factors influencing the LS of hypertensive patients, we included the variables that were significant (*p* < 0.05) in the logistic regression analysis into the RF and ranked the importance of the variables based on the decrease in the Gini coefficient. All data analyses were completed based on the software R 4.3.0, and variables with *p* values <0.05 (two-sided) were considered to indicate a statistically significant difference.

### 2.5 Random forest

RF is a machine-learning method based on classification trees. It is mainly used for classification and regression tasks, which improves the accuracy and stability of predictions by constructing multiple decision trees and integrating the prediction results (36). The basic principle is to use Bootstrap sampling to generate multiple training subsets, each of which independently trains a decision tree, while randomly selecting some features at each node split to increase the diversity of the model. Algorithmically, the RF derives the final prediction by classifying or regressing the prediction results of each decision tree. This method can effectively handle non-linear problems and is resistant to overfitting, as well as being able to handle large numbers of features and data. The model has strong generalization capabilities, and its prediction results are generally superior to other machine algorithms (37).

## 3 Results

### 3.1 Results of univariate analysis

#### 3.1.1 Sociodemographic characteristics and life satisfaction of hypertensive patients

Table 2 shows the descriptive results of the sociodemographic characteristics of hypertensive patients and the results of the univariate analysis of their relationship with LS. Of the 4,197 participants, 2,958 (70.48%) reported being satisfied with their current living situation. The average age of the participants was 82.35 ± 10.67, of which 55.85% were female, 61.19% lived in urban areas, and 53.09% were unmarried. Only 27.18% of the participants had received more than 6 years of education. Most of the participants had a common level of economic status (69.88%). *T*-test and chi-square test showed that age and economic status differed significantly (*p* < 0.05) between different LS levels of hypertensive patients.

**Table 2:** Sociodemographic characteristics and LS of hypertensive patients.

Variables	Total	Satisfied	Not satisfied	Statistic	*P*
Total, *n*(%)	4,197 (100)	2,958 (70.48)	1,239 (29.52)		
Age, Mean ± SD	82.35 ± 10.67	82.76 ± 10.65	81.38 ± 10.67	*t* = 3.82	**<0.001**
Gender, *n*(%)				χ^2^ = 3.63	0.057
Female	2,344 (55.85)	1,680 (56.80)	664 (53.59)		
Male	1,853 (44.15)	1,278 (43.20)	575 (46.41)		
Residence, *n*(%)				χ^2^ = 0.59	0.441
Urban	2,568 (61.19)	1,821 (61.56)	747 (60.29)		
Rural	1,629 (38.81)	1,137 (38.44)	492 (39.71)		
Marital status, *n*(%)				χ^2^ = 0.21	0.648
Not married	2,228 (53.09)	1,577 (53.31)	651 (52.54)		
Married	1,969 (46.91)	1,381 (46.69)	588 (47.46)		
Education level, *n*(%)				χ^2^ = 2.48	0.289
Illiterate	1,736 (41.36)	1,207 (40.80)	529 (42.70)		
0–6 years	1,446 (34.45)	1,041 (35.19)	405 (32.69)		
>6 years	1,015 (24.18)	710 (24.00)	305 (24.62)		
Economic status, *n*(%)				χ^2^ = 353.15	**<0.001**
Good	892 (21.25)	796 (26.91)	96 (7.75)		
Common	2,933 (69.88)	2,024 (68.42)	909 (73.37)		
Poor	372 (8.86)	138 (4.67)	234 (18.89)		

t, *t*-test; χ^2^, Chi-square test; SD, standard deviation; Bolded values, *p* < 0.05.

#### 3.1.2 Health status and life satisfaction of hypertensive patients

The results of the descriptive analysis of the health status of hypertensive patients and the univariate analysis of their relationships with LS are shown in Table 3. Less than half of the participants (47.22%) had a BMI within the normal range. Among participants, abdominal obesity prevalence reached 51.06%, whereas 43.22% considered their health status to be good. Among all participants, 29.88% had visual impairment, 33% had hearing impairment, and 22.06% had cognitive impairment. Most participants were free of diabetes, heart disease, chronic nephritis, hepatitis, cancer, and ADL disability (74.93, 69.76, 93.59, 94.23, 93.11, and 81.65%) and had no depressive and anxiety symptoms (97.43, 77.94%). The results of the chi-square test showed that abdominal obesity, self-rated health, visual impairment, cognitive impairment, and depressive symptoms were significantly different among hypertensive patients with different levels of LS (*p* < 0.05).

**Table 3:** Health status and LS of hypertensive patients.

Variables	Total	Satisfied	Not satisfied	Statistic	*P*
Total, *n*(%)	4,197(100)	2,958(70.48)	1,239(29.52)		
BMI, *n*(%)				χ^2^ = 3.47	0.324
18.5–24	1,982 (47.22)	1,380 (46.65)	602 (48.59)		
<18.5	413 (9.84)	284 (9.60)	129 (10.41)		
24–28	1,316 (31.36)	938 (31.71)	378 (30.51)		
>28	486 (11.58)	356 (12.04)	130 (10.49)		
Abdominal obesity, *n*(%)				χ^2^ = 19.15	**<0.001**
No	2,054 (48.94)	1,383 (46.75)	671 (54.16)		
Yes	2,143 (51.06)	1,575 (53.25)	568 (45.84)		
Self-rated health, *n*(%)				χ^2^ = 651.34	**<0.001**
Good	1,814 (43.22)	1,636 (55.31)	178 (14.37)		
Common	1,739 (41.43)	1,038 (35.09)	701 (56.58)		
Poor	644 (15.34)	284 (9.60)	360 (29.06)		
Visual impairment, *n*(%)				χ^2^ = 17.02	**<0.001**
No	2,943 (70.12)	2,130 (72.01)	813 (65.62)		
Yes	1,254 (29.88)	828 (27.99)	426 (34.38)		
Hearing impairment, *n*(%)				χ^2^ = 1.47	0.225
No	2,812 (67.00)	1,965 (66.43)	847 (68.36)		
Yes	1,385 (33.00)	993 (33.57)	392 (31.64)		
Cognitive impairment, *n*(%)				χ^2^ = 5.46	**0.019**
No	3,271 (77.94)	2,334 (78.90)	937 (75.63)		
Yes	926 (22.06)	624 (21.10)	302 (24.37)		
Diabetes, *n*(%)				χ^2^ = 0.54	0.461
No	3,145 (74.93)	2,226 (75.25)	919 (74.17)		
Yes	1,052 (25.07)	732 (24.75)	320 (25.83)		
Heart disease, *n*(%)				χ^2^ = 0.40	0.525
No	2,928 (69.76)	2,055 (69.47)	873 (70.46)		
Yes	1,269 (30.24)	903 (30.53)	366 (29.54)		
Chronic nephritis, *n*(%)				χ^2^ = 0.00	0.955
No	3,928 (93.59)	2,768 (93.58)	1,160 (93.62)		
Yes	269 (6.41)	190 (6.42)	79 (6.38)		
Hepatitis, *n*(%)				χ^2^ = 0.25	0.617
No	3,955 (94.23)	2,784 (94.12)	1,171 (94.51)		
Yes	242 (5.77)	174 (5.88)	68 (5.49)		
Cancer, *n*(%)				χ^2^ = 0.10	0.757
No	3,908 (93.11)	2,752 (93.04)	1,156 (93.30)		
Yes	289 (6.89)	206 (6.96)	83 (6.70)		
ADL disability, *n*(%)				χ^2^ = 0.06	0.814
No	3,427 (81.65)	2,418 (81.74)	1,009 (81.44)		
Yes	770 (18.35)	540 (18.26)	230 (18.56)		
Anxiety symptoms, *n*(%)				χ^2^ = 0.06	0.811
No	4,089 (97.43)	2,883 (97.46)	1,206 (97.34)		
Yes	108 (2.57)	75 (2.54)	33 (2.66)		
Depressive symptoms, *n*(%)				χ^2^ = 229.50	**<0.001**
No	3,271 (77.94)	2,491 (84.21)	780 (62.95)		
Yes	926 (22.06)	467 (15.79)	459 (37.05)		

χ^2^, Chi-square test; BMI, body mass index; ADL, activities of daily living; Bolded values, *p* < 0.05.

#### 3.1.3 Lifestyles and life satisfaction of hypertensive patients

Table 4 shows the descriptive results of the lifestyle of hypertensive patients and the results of univariate analysis of their relationship with LS. Only a few participants did not eat fruits and vegetables (21.28, 2.81%). Among participants, 68.81% had a light taste, and 38.93% did not sleep for more than 7 h a day. The majority of the participants reported no smoking and drinking habits (70.36, 29.64%). Of all participants, only 39.58% had exercise habits, 72.50% performed physical activities, and 64.69% had social participation. Most participants lived with family members (79.56%), while only 16.70% lived alone. The results of the chi-square test showed that there were significant differences (*p* < 0.05) between the different LS levels of hypertensive patients in terms of fruits, vegetables, taste, sleep duration, exercise, and living arrangements.

**Table 4:** Lifestyles and LS of hypertensive patients.

Variables	Total	Satisfied	Not satisfied	Statistic	*P*
Total, *n*(%)	4,197(100)	2,958(70.48)	1,239(29.52)		
Fruits, *n*(%)				χ^2^ = 55.46	**<0.001**
Not eat	893 (21.28)	568 (19.20)	325 (26.23)		
Eat	2,223 (52.97)	1,541 (52.10)	682 (55.04)		
Almost daily	1,081 (25.76)	849 (28.70)	232 (18.72)		
Vegetables, *n*(%)				χ^2^ = 16.38	**<0.001**
Not eat	118 (2.81)	81 (2.74)	37 (2.99)		
Eat	1,239 (29.52)	820 (27.72)	419 (33.82)		
Almost daily	2,840 (67.67)	2,057 (69.54)	783 (63.20)		
Taste, *n*(%)				χ^2^ = 4.67	**0.031**
Non-light	1,309 (31.19)	893 (30.19)	416 (33.58)		
Light	2,888 (68.81)	2,065 (69.81)	823 (66.42)		
Sleep duration (hours), *n*(%)				χ^2^ = 56.19	**<0.001**
<7	1,634 (38.93)	1,047 (35.40)	587 (47.38)		
7–9	1,883 (44.87)	1,385 (46.82)	498 (40.19)		
>9	680 (16.20)	526 (17.78)	154 (12.43)		
Smoking, *n*(%)				χ^2^ = 1.36	0.243
Non-smoker	2,953 (70.36)	2,097 (70.89)	856 (69.09)		
Smoker	1,244 (29.64)	861 (29.11)	383 (30.91)		
Drinking, *n*(%)				χ^2^ = 0.49	0.485
Non-drinker	3,140 (74.82)	2,222 (75.12)	918 (74.09)		
Drinker	1,057 (25.18)	736 (24.88)	321 (25.91)		
Exercise, *n*(%)				χ^2^ = 29.39	**<0.001**
No	2,536 (60.42)	1,709 (57.78)	827 (66.75)		
Yes	1,661 (39.58)	1,249 (42.22)	412 (33.25)		
Physical activity, *n*(%)				χ^2^ = 0.92	0.337
No	1,154 (27.50)	826 (27.92)	328 (26.47)		
Yes	3,043 (72.50)	2,132 (72.08)	911 (73.53)		
Social participation, *n*(%)				χ^2^ = 0.66	0.415
No	1,482 (35.31)	1,056 (35.70)	426 (34.38)		
Yes	2,715 (64.69)	1,902 (64.30)	813 (65.62)		
Living arrangements, *n*(%)				χ^2^ = 29.52	**<0.001**
Family member	3,339 (79.56)	2,417 (81.71)	922 (74.41)		
Alone	701 (16.70)	437 (14.77)	264 (21.31)		
Institution	157 (3.74)	104 (3.52)	53 (4.28)		

χ^2^, Chi-square test; Bolded values, *p* < 0.05.

### 3.2 Results of multivariable analysis

Table 5 shows the results of multifactorial analysis of LS of hypertensive patients. Regression analysis showed that gender, economic status, self-rated health, hearing impairment, heart disease, depressive symptoms, fruits, sleep duration, and living arrangements were independent factors influencing the LS of hypertensive patients (*p* < 0.05). Compared with females, males had a higher risk of poor LS (OR = 1.33, 95% CI: 1.08 ~ 1.64). Patients with poor economic status had a 7.13-fold higher risk of reporting low LS than those with good economic status (OR = 7.13, 95% CI: 5.10 ~ 9.96). Patients with poor self-rated health were 8.74 times more likely to report low LS than those with good self-rated health (OR = 8.74, 95% CI: 6.84 ~ 11.18). Hypertensive patients who had hearing impairment had higher LS than those with normal hearing (OR = 0.79, 95% CI: 0.66 ~ 0.96). Hypertensive patients with heart disease reported a 25% lower risk of low LS compared to those without heart disease (OR = 0.75, 95% CI:0.62 ~ 0.90), while patients with depressive symptoms reported a 90% increased risk of low LS compared to those who did not show depressive symptoms (OR = 1.90, 95% CI:1.59 ~ 2.27). Patients who ate fruit almost daily and slept more than 9 h were more likely to report high LS (OR = 0.74, 95% CI:0.58 ~ 0.95; OR = 0.64, 95% CI: 0.51 ~ 0.82). Compared with patients living with family members, those living in institutions or living alone had lower LS (OR = 1.71, 95% CI: 1.14 ~ 2.58; OR = 1.64, 95% CI:1.30 ~ 2.05).

**Table 5:** Multivariable analysis of LS of hypertensive patients.

Variables	*β*	S.E	Z	*P*	OR (95%CI)
Age	−0.01	0.01	−1.68	0.092	0.99 (0.98 ~ 1.00)
Gender					
Female					1.00 (Reference)
Male	0.28	0.11	2.68	**0.007**	1.33 (1.08 ~ 1.64)
Residence					
Urban					1.00 (Reference)
Rural	−0.01	0.09	−0.09	0.932	0.99 (0.84 ~ 1.18)
Marital status					
Not married					1.00 (Reference)
Married	0.02	0.11	0.20	0.842	1.02 (0.83 ~ 1.26)
Education level					
Illiterate					1.00 (Reference)
0–6 years	−0.09	0.10	−0.83	0.406	0.92 (0.75 ~ 1.12)
>6 years	0.16	0.13	1.19	0.234	1.17 (0.90 ~ 1.52)
Economic status					
Good					1.00 (Reference)
Common	1.04	0.13	8.33	**<0.001**	2.84 (2.22 ~ 3.63)
Poor	1.96	0.17	11.48	**<0.001**	7.13 (5.10 ~ 9.96)
BMI					
18.5–24					1.00 (Reference)
<18.5	0.04	0.14	0.27	0.788	1.04 (0.79 ~ 1.36)
24–28	0.04	0.16	0.25	0.803	1.04 (0.76 ~ 1.41)
>28	−0.03	0.19	−0.15	0.880	0.97 (0.67 ~ 1.40)
Abdominal obesity					
No					1.00 (Reference)
Yes	−0.12	0.09	−1.32	0.187	0.89 (0.75 ~ 1.06)
Self-rated health					
Good					1.00 (Reference)
Common	1.66	0.10	16.89	**<0.001**	5.25 (4.33 ~ 6.36)
Poor	2.17	0.13	17.27	**<0.001**	8.74 (6.84 ~ 11.18)
Visual impairment					
No					1.00 (Reference)
Yes	0.10	0.09	1.07	0.284	1.10 (0.92 ~ 1.32)
Hearing impairment					
No					1.00 (Reference)
Yes	−0.23	0.09	−2.43	**0.015**	0.79 (0.66 ~ 0.96)
Cognitive impairment					
No					1.00 (Reference)
Yes	0.13	0.10	1.23	0.219	1.13 (0.93 ~ 1.39)
Diabetes					
No					1.00 (Reference)
Yes	−0.12	0.10	−1.16	0.245	0.89 (0.73 ~ 1.08)
Heart disease					
No					1.00 (Reference)
Yes	−0.29	0.10	−3.01	**0.003**	0.75 (0.62 ~ 0.90)
Chronic nephritis					
No					1.00 (Reference)
Yes	0.13	0.27	0.49	0.623	1.14 (0.67 ~ 1.95)
Hepatitis					
No					1.00 (Reference)
Yes	0.26	0.32	0.80	0.422	1.29 (0.69 ~ 2.42)
Cancer					
No					1.00 (Reference)
Yes	0.10	0.23	0.43	0.664	1.10 (0.71 ~ 1.72)
ADL disability					
No					1.00 (Reference)
Yes	−0.04	0.12	−0.35	0.729	0.96 (0.76 ~ 1.21)
Anxiety symptoms					
No					1.00 (Reference)
Yes	−0.13	0.24	−0.57	0.570	0.87 (0.55 ~ 1.39)
Depressive symptoms					
No					1.00 (Reference)
Yes	0.64	0.09	7.07	**<0.001**	1.90 (1.59 ~ 2.27)
Fruits					
Not eat					1.00 (Reference)
Eat	−0.07	0.10	−0.66	0.511	0.94 (0.77 ~ 1.14)
Almost daily	−0.30	0.13	−2.32	**0.020**	0.74 (0.58 ~ 0.95)
Vegetables					
Not eat					1.00 (Reference)
Eat	−0.03	0.25	−0.12	0.901	0.97 (0.59 ~ 1.58)
Almost daily	−0.04	0.25	−0.16	0.871	0.96 (0.59 ~ 1.56)
Taste					
Non-light					1.00 (Reference)
Light	−0.15	0.08	−1.76	0.078	0.86 (0.73 ~ 1.02)
Sleep duration(hours)					
7–9					1.00 (Reference)
<7	−0.13	0.09	−1.50	0.134	0.88 (0.74 ~ 1.04)
>9	−0.44	0.12	−3.57	**<0.001**	0.64 (0.51 ~ 0.82)
Smoking					
Non-smoker					1.00 (Reference)
Smoker	0.01	0.11	0.12	0.905	1.01 (0.82 ~ 1.25)
Drinking					
Non-drinker					1.00 (Reference)
Drinker	0.03	0.10	0.31	0.759	1.03 (0.84 ~ 1.27)
Exercise					
No					1.00 (Reference)
Yes	−0.16	0.09	−1.87	0.061	0.85 (0.72 ~ 1.01)
Physical activity					
No					1.00 (Reference)
Yes	−0.04	0.10	−0.36	0.716	0.97 (0.80 ~ 1.17)
Social participation					
No					1.00 (Reference)
Yes	−0.15	0.09	−1.62	0.105	0.86 (0.71 ~ 1.03)
Living arrangements					
Family member					1.00 (Reference)
Alone	0.49	0.12	4.25	**<0.001**	1.64 (1.30 ~ 2.05)
Institution	0.54	0.21	2.57	**0.010**	1.71 (1.14 ~ 2.58)

OR, Odds Ratio; CI, Confidence Interval; BMI, body mass index; ADL, activities of daily living; Bolded values, *p* < 0.05.

### 3.3 Results of random forest

**Figure 2:**
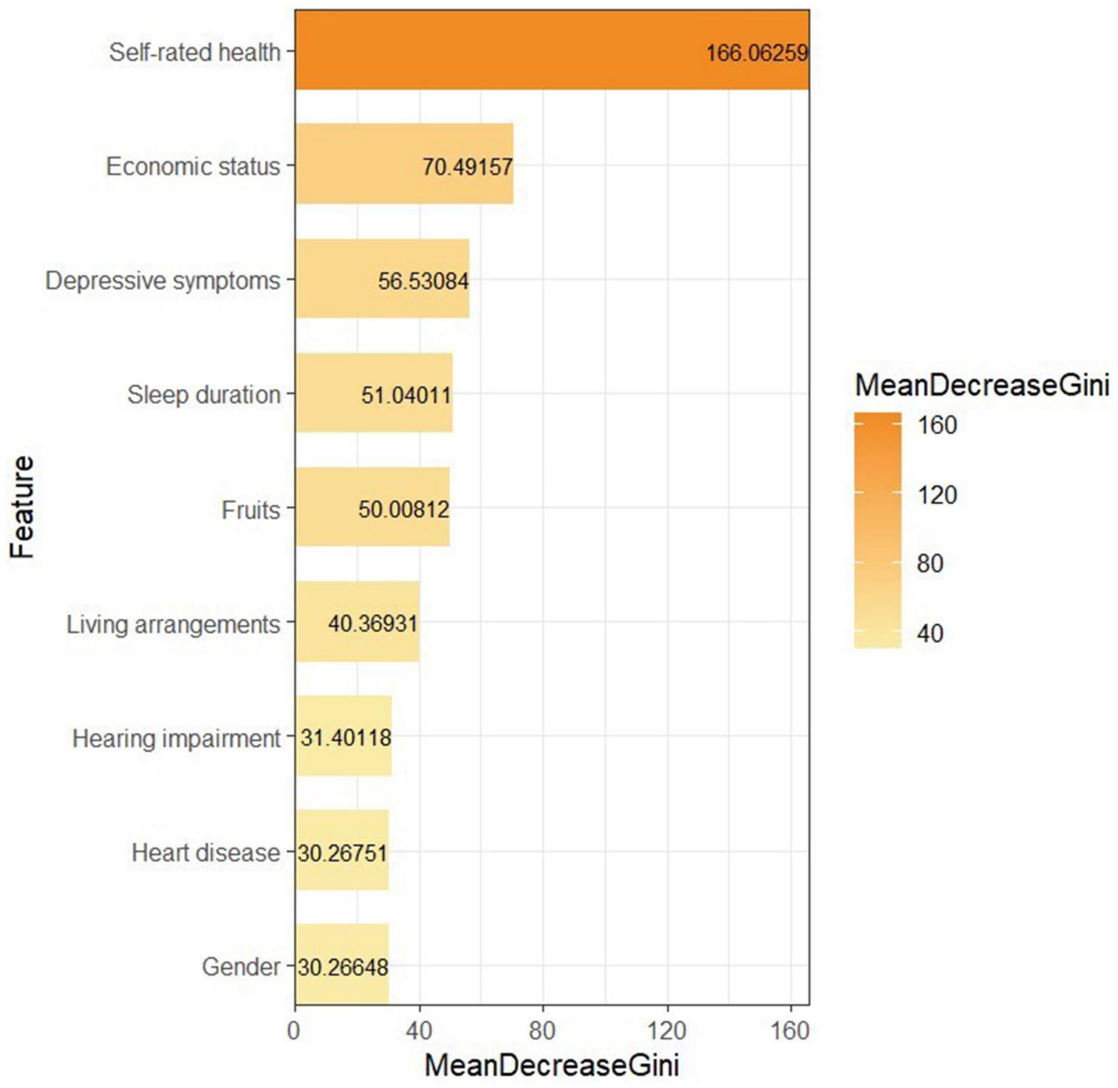
Ranking of variable importance in random forest.

To further assess the relative importance of the influencing factors, we included the significant influencing variables obtained from the multivariate analysis into the RF and sorted them from high to low according to MeanDecreaseGini. The results are shown in Figure 2 (where the parameters of the RF are ntree = 500 and mtry = 3). The influencing factors were, in descending order, self-rated health, economic status, depressive symptoms, sleep duration, fruits, living arrangements, hearing impairment, heart disease, and gender.

## 4 Discussion

Using the nationally representative dataset, this study analyzed the prevalence and influencing factors of life dissatisfaction among Chinese older adults with hypertension and developed the RF to rank the importance of these factors. Overall, among all participants, 29.52% of hypertensive patients reported being dissatisfied with their lives. The study showed that the LS of hypertensive patients is influenced by variables in multiple dimensions. The final RF results showed that the key factors influencing the LS of hypertensive patients were, in order of importance, self-rated health, economic status, depressive symptoms, sleep duration, fruits, living arrangements, hearing impairment, heart disease, and gender.

Similar to the result of another study (38), the better the self-rated health, the higher the LS of the older adults. Self-rated health can be understood as a brief statement about how different aspects of health are combined within an individual’s perception (39). Due to its inclusiveness and non-definitive nature, it has been widely used as one of the most commonly used indicators of health status in various studies (40–42). Recent research has shown that subjective attitude towards health status can indirectly reflect an individual’s satisfaction with their current life (43). For hypertensive patients, the better their self-rated health, the higher their satisfaction with their health, and the less subjectively disturbed by the disease than those with poor self-rated health. As a result, they tend to have a better psychological state and a positive attitude towards life, so they are able to optimistically face the adverse effects of hypertension and other complications in their lives.

Our study found that there is a significant correlation between the economic situation and LS of hypertensive patients, which is consistent with the results of a previous study (44). Hypertension is a chronic disease that requires long-term treatment and control. Patients with hypertension need to take medication continuously, purchase medical devices, and undergo regular medical examinations. At the same time, they also need lifestyle adjustments such as a healthy diet and regular exercise. Financial support plays a significant role in all of these aspects. Poor economic status may mean that patients have limited access to high-quality medical services and daily care, which in turn leads to physical backwardness and psychological impairment in older adults. Some of the medical expenses that are necessary for hypertension will further increase the financial burden on poor people, thus reducing their LS.

The study also demonstrated a significant correlation between depressive symptoms and low LS in older adults with hypertension, which is also supported by a previous study (45). Depression is a psychological state of sadness, helplessness, and melancholy, with mood changes and somatic symptoms being its most common manifestations (46). Patients with depressive symptoms often lack positive emotions, lose enthusiasm and interest in life, and therefore have a reduced perception of well-being, showing a decrease in LS. At the same time, depression can be accompanied by sleep disorders and loss of appetite, which exacerbate physical fatigue and lead to a decline in functions such as the digestive and immune systems. A prolonged depressive state can cause serious damage to mental health and various bodily functions, thereby increasing the risk of developing various chronic diseases. A recent study provides preclinical evidence that lycopene may serve as a potential antidepressant, which provides an effective way to develop novel antidepressant therapies and improve the prognosis and LS of hypertensive patients (47).

In our study, patients with long sleep duration were more likely to report high LS, which may resonate with previous studies (48, 49). As one of the response indicators of sleep quality, there is a strong bidirectional relationship between sleep duration and LS (50). Studies have shown that adequate sleep helps to restore body functions and maintain metabolism, thus promoting the improvement of overall health (51, 52). For hypertensive patients, an appropriate amount of sleep can reduce their risk of secondary cardiovascular and cerebrovascular diseases and other age-related diseases, helping them control blood pressure and improve their prognosis (53). Sleep quality is also closely related to the gut microbiota. Gut microbes have been demonstrated to regulate blood pressure through immune, neural, and endocrine mechanisms (54, 55). Longer sleep durations provide optimal conditions for these microbes to influence bodily functions, which, to some extent, alleviates the disease burden of hypertensive patients. Meanwhile, adequate sleep also facilitates the regulation of some mood-related hormones (51), thereby alleviating negative emotions such as anger, anxiety, and tension and promoting mental health. Additionally, sleep can relieve physical and mental fatigue and increase an individual’s vitality. These conclusions all suggest that a longer sleep duration can significantly improve the LS of hypertensive patients. A recent study by Rachmawati et al. (56) demonstrated a significant improvement in sleep quality through hyperbaric oxygen therapy. This suggests a viable approach for managing blood pressure and preventing low life satisfaction in patients with hypertension.

We found that patients who ate fruit every day had a higher level of LS. Fruit intake is an important part of a healthy lifestyle. Numerous studies have shown that fruit has an undeniable effect on promoting physical health (57–60). Fruits are rich in a variety of vitamins, minerals, and other essential nutrients for the body, which can fight oxidation, promote digestion, and lower cholesterol, thereby helping hypertensive patients control blood pressure and prevent the occurrence of other chronic cardiovascular diseases (61). At the same time, eating fruits can also promote the improvement of mental health, which is also related to the large amount of vitamin B, vitamin C, antioxidants, carotenoids, and other substances in fruits (62). These substances may relieve stress, anxiety, tension, and depression by lowering the level of stress hormones in the body so as to promote the level of mental health of individuals (57, 62). Therefore, fruit plays a significant role in regulating blood pressure and enhancing overall life satisfaction. It is worth noting that the mechanism and effect of fruit on blood pressure regulation can vary depending on the type of fruit. Flavonoid-rich fruits such as citrus can inhibit the activity of angiotensin-converting enzyme (ACE) and improve vascular endothelial function (63, 64). They can also help regulate gut microbiota, contributing to blood pressure regulation. Berry fruits are rich in anthocyanins, which can activate endothelial nitric oxide synthase (eNOS) to promote vasodilation. They can also change the diversity and composition of gut microorganisms, promote the production of short-chain fatty acids (SCFAs), and thus exert a blood pressure-lowering effect (65, 66). In addition to the above mechanisms, fruits rich in dietary fiber, such as drupes, can improve the elasticity of blood vessel walls, thereby reducing blood vessel resistance and improving insulin sensitivity (67, 68). Moreover, the timing of fruit intake also has a different effect on blood pressure regulation. Eating fruit before meals can help hypertensive patients take better advantage of the satiating effects of dietary fiber, leading to reduced salt and fat intake. This can help them lose weight and protect cardiovascular function, which are all crucial measures to control blood pressure (69).

In addition to the above-mentioned key factors, there are a number of other factors that also played an important role in our study. For example, our study proved that participants living with their families have higher LS. This is similar to previous research (70). We speculate that the possible reason may be influenced by the Chinese family culture of filial piety and love for older adults. Family members can provide emotional support for older adults with hypertension, helping them to actively cope with difficulties and challenges in life through companionship, encouragement, and comfort. They may do so by taking care of their diets and daily lives and accompanying them to participate in social activities such as walking, fitness, and gatherings, which not only provides them with more opportunities to exercise but also makes them feel cared for and warmed up, thereby reducing feelings of loneliness and anxiety.

Hearing impairment is defined as a decrease in hearing thresholds at different frequencies, unilaterally or bilaterally (71). It has a huge negative impact on daily life. However, our study found that respondents with hearing impairment had higher LS, which is inconsistent with previous research results (72, 73). We speculate that although people with hearing impairment often feel inadequate in daily life and social interactions due to difficulties in speech recognition and output, to some extent, such impairments can help them isolate themselves from the interference of unpleasant sounds and improve their concentration. At the same time, they also enjoy higher social welfare and subsidies and more social care. These factors may lead to higher LS.

Similarly, we found that respondents with heart disease were more likely to report satisfaction with life, which may be related to a range of secondary effects of the disease. While heart disease places a heavy burden on the health of patients, it also increases their appreciation of healthy living. They tend to adopt more meticulous daily care and a more cautious lifestyle, such as careful regulation of diet and maintenance of calmness, as well as intentional avoidance of unhealthy lifestyles, which to some extent improves their physical and mental health. In addition, the care and support of society may also give them a greater sense of well-being and belonging.

Additionally, we found that gender also has a large impact on LS. Current studies are divided on this view (74, 75). In our study, male older adults with hypertension had lower LS than females. The reason may be related to the feudal social culture of “male goes out to work while female looks after the house” in the old society. In family life, the male’s role as a “pillar” usually leads them to bear heavier financial responsibilities and face greater mental pressure. After falling ill, their physical function declines, their sense of social value and self-esteem decreases, while psychological pressure further increases, which in turn leads to a decline in LS.

## 5 Limitation

Undeniably, there are still some limitations in our study that should be acknowledged. First, this study is a cross-sectional study based on the CLHLS database, making it impossible to establish the in-depth causal relationship between these influencing factors and LS. Second, most of the data for the variables came from self-reported questions, and we have to consider the impact of recall bias and subjective factors of the participants on the authenticity of the data. In addition, due to the limitations of the survey content of the database, many variables that may be important were not included in this study. For example, the intake of some medications also has a direct effect on blood pressure, which may in turn affect LS of hypertensive patients, such as those associated with thyroid disorders (76, 77). Further consideration of rich variables and diversity of measurement indicators is worth undertaking in future research.

## 6 Conclusion

In summary, this study used cross-sectional data published by CLHLS 2018 to analyze the influencing factors of LS in older adults with hypertension in China from three aspects: sociodemographic characteristics, health status, and lifestyles. The RF was used further to rank the importance of each significant influencing factor. These findings are of great guiding significance for the prevention and intervention of life dissatisfaction among older adults with hypertension. We advocate that policymakers and relevant health practitioners should focus on hypertensive patients with poor self-rated health, poor economic status, depressive symptoms, short sleep duration, no fruit consumption, living alone or in an institution, hearing impairment, and heart disease, and female hypertensive patients when formulating policies and providing clinical care.

## Data Availability

The datasets presented in this study can be found in online repositories. The names of the repository/repositories and accession number(s) can be found in the article/supplementary material.

## Footnotes

^1^ https://opendata.pku.edu.cn/dataverse/CHADS
